# A Lower Maternal Cortisol-to-Cortisone Ratio Precedes Clinical Diagnosis of Preterm and Term Preeclampsia by Many Weeks

**DOI:** 10.1210/jc.2018-02312

**Published:** 2019-02-15

**Authors:** Nimesh A Jayasuriya, Alice E Hughes, Ulla Sovio, Emma Cook, D Stephen Charnock-Jones, Gordon C S Smith

**Affiliations:** 1 University of Glasgow School of Medicine, Glasgow, United Kingdom; 2 Centre for Trophoblast Research, Department of Physiology, Development and Neuroscience, University of Cambridge, Cambridge, United Kingdom; 3 Department of Physiology, Development and Neuroscience, Centre for Trophoblast Research, University of Cambridge, Cambridge, United Kingdom

## Abstract

**Context:**

Previous studies have shown reduced placental levels of 11-*β*-hydroxysteroid dehydrogenase type 2 (11*β*HSD2) in preeclampsia (PE). However, it is unknown if the maternal cortisol-to-cortisone ratio is predictive of placental complications of pregnancy.

**Objective:**

To determine the relationship between the maternal serum cortisol-to-cortisone ratio at different stages of pregnancy and the risk of PE or fetal growth restriction (FGR).

**Design:**

Women from the Pregnancy Outcome Prediction Study experiencing PE (n = 194) or FGR (n = 185), plus a random sample of healthy controls (n = 279), were studied. Steroids were measured at ∼12, ∼20, ∼28, and ∼36 weeks of gestational age (wkGA). Separate analyses were performed for outcomes with term or preterm delivery. Associations were modeled using logistic regression.

**Results:**

At 28 wkGA, the cortisol-to-cortisone ratio was negatively associated (OR per 1 SD increase, 95% CI)] with preterm PE (OR 0.33, 95% CI 0.19 to 0.57), term PE (OR 0.61, 95% CI 0.49 to 0.76), and preterm FGR (OR 0.50, 95% CI 0.29 to 0.85). At 36 wkGA, the cortisol-to-cortisone ratio was negatively associated with term PE (OR 0.42, 95% CI 0.32 to 0.55) but not term FGR (OR 1.07, 95% CI 0.87 to 1.31). Associations were not materially affected by adjustment for maternal characteristics.

**Conclusions:**

A lower maternal serum cortisol-to-cortisone ratio precedes clinical manifestation of PE and preterm FGR by many weeks, despite previous reports of reduced levels of placental 11*β*HSD2 in these conditions. Our observations implicate enhanced maternal 11*β*HSD2 activity or reduced 11*β*HSD type 1 activity in the pathophysiology of PE.

Preeclampsia (PE) and fetal growth restriction (FGR) are poorly understood conditions of pregnancy associated with substantial perinatal morbidity and mortality. PE is a disorder of placental origin, characterized by hypertension and proteinuria that develops during the antenatal or immediate postpartum period (1). It affects between 2% and 8% of pregnancies and is a major cause of direct maternal death worldwide (2, 3). It is often accompanied by FGR, defined as a fetus not reaching its genetically determined growth potential (4) as a result of poor placental function. Currently, there are no effective management options available, apart from delivery, and accurate early prediction of the disease is not possible.

One marker of placental function that might be affected in PE is the maternal serum cortisol-to-cortisone ratio (5). 11-*β*-Hydroxysteroid dehydrogenase type 2 (11*β*HSD2) is a steroid enzyme highly expressed in the placenta and with the metabolization of cortisol to cortisone, protects the fetus from exposure to excessive maternal cortisol during pregnancy, whereas 11*β*HSD type 1 11*β*HSD1 (11BHSD1) performs the reverse of 11*β*HSD2 and reactivates cortisol by converting cortisone to cortisol (6). Several studies have reported that both PE and FGR are associated with reduced levels of placental 11*β*HSD2 (7–15) and reduced expression and activity of placental 11*β*HSD1 have been demonstrated in cases of small for gestational age (SGA) (16, 17). Reduced 11*β*HSD2 activity would be expected to result in an increased maternal serum cortisol-to-cortisone ratio; hence, these findings suggest that the maternal serum cortisol-to-cortisone ratio may be used as a predictor or marker of disease. A small number of studies previously addressed this question and paradoxically, reported that women with PE exhibited lower cortisol-to-cortisone ratios, suggesting increased maternal 11*β*HSD2 activity (18–20). However, these analyses were limited to samples obtained following diagnosis and consisted of studies that had not included >14 cases of confirmed PE and 14 cases of confirmed FGR per study. The aim of the current study was to investigate the relationship between maternal cortisol and cortisone levels with PE and FGR before the onset of disease, using measurements taken at different time points in pregnancy in a large number of optimally characterized cases and controls.

## Materials and Methods

### Study design

We used a case-cohort design from the Pregnancy Outcome Prediction (POP) Study. Details of the study are provided elsewhere (21), but in brief, it was a prospective cohort study that collected blood, ultrasound scan, and pregnancy outcome data from 4212 nulliparous women at ∼12, ∼20, ∼28, and ∼36 weeks of gestational age (wkGA) between 14 January 2008 and 31 July 2012 (22, 23). The case cohort included 379 women with cases of PE and FGR and a random sample of the total cohort (n = 325) (24). Out of the 325 women in the random subcohort, 279 did not experience any adverse outcome and are referred to as “healthy.”

### Definitions of outcomes studied

For this analysis, we studied women with PE or FGR and divided them into preterm and term subgroups based on gestational age at delivery (<37 wkGA and ≥37 wkGA, respectively). PE was diagnosed according to the American College of Obstetricians and Gynecologists (ACOG) 2013 Guidelines (25), and the cases in this analysis included all severe PE at term; nonsevere, nonsuperimposed PE at term; and all preterm PE. PE cases with preterm delivery were compared with all of the women in the subcohort who did not experience PE leading to preterm birth. Term PE cases were compared with women from the subcohort who delivered at term without experiencing any type of PE. Preterm FGR was defined as SGA <10th customized percentile (26) with delivery <37 wkGA. Preterm FGR cases were compared with women in the subcohort who did not deliver a preterm infant with FGR. We excluded preterm cases of PE from analyses of preterm FGR. FGR at term was defined as severe SGA (birth weight less than third customized percentile) or SGA with growth restriction (birth weight <10th customized percentile and reduced growth velocity of the abdominal circumference on serial ultrasound scans) with delivery ≥37 wkGA. Term FGR cases were compared with women in the subcohort who delivered an infant at term without FGR. In addition to preterm births, we excluded all types of term PE from analyses of term FGR. Exclusion of preterm births from the analysis of term outcome was done, as women delivering preterm are necessarily not at risk for complications at term, and the exclusion of PE from the analysis of FGR was made to separate better the two complications, which can be overlapping, especially at preterm gestational ages.

Outcomes were collected from paper-based hospital records and relevant electronic databases. Maternal characteristics [age, marital status, ethnicity, smoking, age at leaving full-time education (FTE)] were self-reported, except for height and body mass index (BMI), which were measured at the time of recruitment, and deprivation, which was measured using the Index of Multiple Deprivation 2007 (27), calculated from the woman’s postcode.

### Steroid measurements

Steroid levels were measured in maternal serum at Metabolon, Inc. (Durham, NC) by nontargeted, ultra-HPLC and tandem mass spectrometry. Samples were run in batches of 36, and all batches contained an equal proportion of cases and controls. All samples from a given participant were run in the same batch. Peaks were quantified using area under the curve of primary mass spectrometry ions. Missing values were assumed to be the result of falling below the detection sensitivity and thus, were imputed with the minimum detection value based on each metabolite. In the entire POP Study, where 3196 samples were successfully processed, only one cortisol value and two cortisone values were missing (<0.1%). For this analysis, only one woman in the random subcohort had a missing cortisol and cortisone value, and this was at 12 wkGA. To adjust for instrument batch effects for each run day, the raw ion counts for each steroid were divided by the median value for the run day. Internal standards were not available for all metabolites, so values were not quantified in standard International System of Units. Hence, cortisol and cortisone levels were expressed as multiples of the median (MoM) (28).

### Statistical analyses

The cortisol-to-cortisone ratio for a given sample was generated by division of the MoM for cortisol by the MoM for cortisone. Initial descriptive analyses were then conducted by the summarization of cortisol, cortisone, and the cortisol-to-cortisone ratio for each time of measurement (12, 20, 28, and 36 wkGA) in healthy pregnancies and women with PE or FGR. Linear regression analyses for cortisone against cortisol were performed in cases and controls, and *P* values for interaction between these groups were calculated. We excluded the 36-wkGA measurement from all the analyses of preterm delivery. The MoM values for the given hormone and their ratio at the given time of sampling were converted to Z scores, using the subcohort as the reference. Unadjusted logistic regressions were performed to analyze their associations with the outcomes. These analyses were repeated, adjusting for maternal characteristics (age at recruitment, age left FTE, height, BMI, smoking status, deprivation, ethnicity, marital status). Missing values were imputed using the mode for categorical variables and mean for continuous variables.

Time-to-event analyses (29) were then conducted for term PE from the 36-wkGA measurement onward and preterm PE from the 28-wkGA measurement onward. We studied the whole case-cohort sample, weighting the noncases of the comparator group by the inverse of the sampling fraction (30). We compared the cumulative incidence of PE between women with a cortisol-to-cortisone ratio in the first decile (using a threshold derived from the random subcohort) with women in the second to 10th deciles. Delivery without PE was considered a competing risk.

Finally, we performed a sensitivity analysis confined to women, where it was documented that they had not received steroids antenatally (either for fetal lung maturation or medical conditions, such as asthma). As the research database only started recording information on whether women had received steroids to accelerate fetal lung maturation from 27 November 2009 onward, the majority of women excluded in the sensitivity analysis were omitted because the data were absent, rather than they were documented as receiving steroids. Where the apparent statistical significance of an association between an outcome and the cortisol-to-cortisone ratio differed in the sensitivity analysis, we tested for an interaction between the ratio and antenatal steroid use to determine if there was a true difference between the groups or whether the *P* value became nonsignificant as a result of the reduced sample size in the subgroup.

### Statistical software

All analyses were performed in Stata version 14.0 (StataCorp, College Station, TX).

### Ethics approval

All patients gave their informed, written consent to participate, and the study was approved by the Cambridgeshire 2 Research Ethics Committee (Reference Number 07/H0308/163).

## Results

### Maternal characteristics

There were 878, 873, 854, and 591 women with steroid measurements at mean (±SD) gestational ages 89 (±6), 143 (±3), 198 (±3), and 254 (±3) days, respectively. These are reported as measurements at 12, 20, 28, and 36 wkGA. Maternal characteristics of the women studied are summarized in Table 1. There were 29 cases of preterm PE, 160 cases of PE at term, 25 cases of preterm FGR, and 165 cases of FGR at term in the case cohort for the main analysis. The sensitivity analysis included 12 cases of preterm PE, 123 cases of PE at term, 15 cases of preterm FGR, and 125 cases of FGR at term, and 251 women remained in the random subcohort.

**Table 1.** Maternal Characteristics and Birth Outcomes in Women With Healthy Pregnancies, PE, and FGR

	Healthy^*a*^	PE^*b*^		FGR^*c*^	
		Preterm	Term	Preterm	Term
n (% of case cohort)	279 (30.2)	29 (3.1)	165 (17.9)	25 (2.7)	160 (17.3)
Maternal characteristics					
Age, y, mean (SD)	30 (5)	28 (6)	30 (6)	30 (5)	30 (6)
Age left FTE, y, mean (SD)	21 (4)	20 (4)	20 (4)	20 (3)	21 (4)
Missing, n (%)	1 (0.3)	1 (3.4)	5 (3.0)	0 (0.0)	3 (1.9)
Height, cm, mean (SD)	165 (6)	162 (8)	164 (6)	165 (8)	165 (7)
BMI, kg/m^2^, mean (SD)	24.9 (4.7)	29.7 (5.9)	27.8 (6.3)	25.0 (4.9)	25.6 (5.0)
Booking MAP, mmHg, mean (SD)	78 (9)	87 (10)	83 (10)	81 (9)	79 (8)
Missing, n (%)	9 (3.2)	0 (0.0)	4 (2.4)	1 (4.0)	6 (3.8)
Married, n (%)	203 (72.8)	22 (75.9)	105 (63.6)	16 (64.0)	98 (61.3)
Smoker, n (%)	13 (4.7)	1 (3.5)	6 (3.6)	3 (12.0)	29 (18.1)
Alcohol, n (%)	10 (3.6)	0 (0.0)	7 (4.2)	1 (4.0)	7 (4.4)
Deprivation quartile, n (%)					
1 (lowest)	60 (21.5)	8 (27.6)	41 (24.8)	3 (12.0)	42 (26.3)
2	81 (29.0)	2 (6.9)	38 (23.0)	6 (24.0)	27 (16.9)
3	57 (20.4)	14 (48.3)	40 (24.2)	6 (24.0)	41 (25.6)
4 (highest)	71 (25.4)	5 (17.2)	38 (23.0)	9 (36.0)	43 (26.9)
Missing	10 (3.6)	0 (0.0)	8 (4.8)	1 (4.0)	7 (4.4)
White ethnicity, n (%)	263 (94.3)	26 (89.7)	157 (95.2)	21 (84.0)	151 (94.4)
Nonwhite ethnicity, n (%)	11 (3.9)	2 (6.9)	7 (4.2)	4 (16.0)	7 (4.4)
Missing	5 (1.8)	1 (3.4)	1 (0.6)	0 (0.0)	2 (1.3)
Characteristics of delivery, n (%)					
Mode of delivery					
Vaginal	140 (50.2)	7 (24.1)	36 (21.8)	6 (24.0)	94 (58.8)
Assisted vaginal	58 (20.8)	0 (0.0)	51 (30.9)	5 (20.0)	30 (18.8)
Intrapartum caesarean	54 (19.4)	3 (10.3)	55 (33.3)	1 (4.0)	16 (10.0)
Prelabor caesarean	25 (9.0)	19 (65.6)	22 (13.3)	13 (52.0)	20 (12.5)
Missing	2 (0.7)	0 (0.0)	1 (0.6)	0 (0.0)	0 (0.0)
Labor induced	88 (31.5)	7 (24.1)	106 (64.2)	4 (16.0)	54 (33.8)

Data are expressed as means (±SD) or n (%) with number of missing values below each characteristic. Where there is no missing category, data were complete.

Abbreviation: MAP, mean arterial blood pressure.

^a^ Women without FGR, PE, gestational diabetes, or spontaneous preterm delivery.

^b^ Diagnosed according to ACOG 2013 Guidelines (25) and divided into preterm (delivery <37 wkGA) and term (delivery ≥37 wkGA) outcomes. Cases of PE include all severe and nonsuperimposed, nonsevere PE at term, and all preterm PE.

^c^ Divided into preterm (delivery <37 wkGA) and term (delivery ≥37 wkGA) outcomes. Cases of FGR at term include severe SGA (birth weight less than third customized percentile) and SGA with growth restriction (birth weight <10th customized percentile and reduced growth velocity of the abdominal circumference on serial ultrasound scans) without PE. Cases of preterm FGR include SGA (birth weight <10th customized percentile) with preterm delivery and without PE. Maternal characteristics and the summary of cortisol and cortisone levels were summarized for cases and healthy women as a result of the small overlap in cases in the random subcohort.

### Patterns of cortisol, cortisone, and cortisol-to-cortisone ratio throughout pregnancy

**Figure 1.**
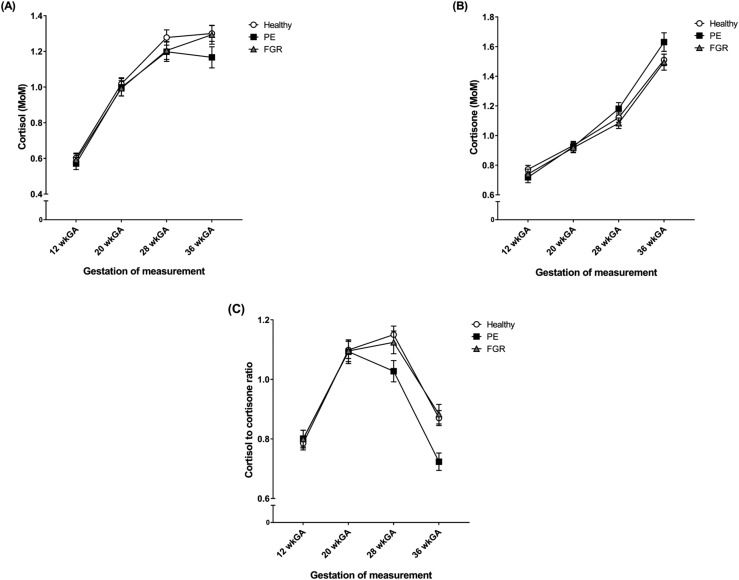
Mean with 95% CIs for (A) cortisol, (B) cortisone, and the (C) cortisol-to-cortisone ratio at 12, 20, 28, and 36 wkGA in healthy pregnancies, women that developed PE, and women who delivered an FGR infant. Cortisol and cortisone are reported as MoM. Healthy pregnancies included women who did not have FGR, PE, gestational diabetes, or spontaneous preterm delivery. Cases of PE were defined by the ACOG 2013 Guidelines (25) and included all severe and nonsevere, nonsuperimposed PE at term and all preterm PE. Cases of term FGR included severe SGA (birth weight less than third customized percentile at term) and SGA with growth restriction (birth weight <10th customized percentile at term with reduced growth velocity of the abdominal circumference on serial ultrasound scans) without PE. Cases of preterm FGR included SGA with preterm delivery (birth weight <10th customized percentile and delivery before 37 completed wkGA) without PE.

In healthy women, cortisol and cortisone both increased as pregnancy progressed [Fig. 1(A) and 1(B), respectively], whereas the cortisol-to-cortisone ratio showed an upward trend between 12 and 20 wkGA, plateaued until 28 wkGA, and then fell at 36 wkGA [Fig. 1(C)]. This pattern was similar in cases of FGR, but in PE, the cortisol-to-cortisone ratio started to decline at 28 wkGA onward [Fig. 1(C)].

### Associations among cortisol, cortisone, and cortisol-to-cortisone ratio with PE and FGR

**Figure 2.**
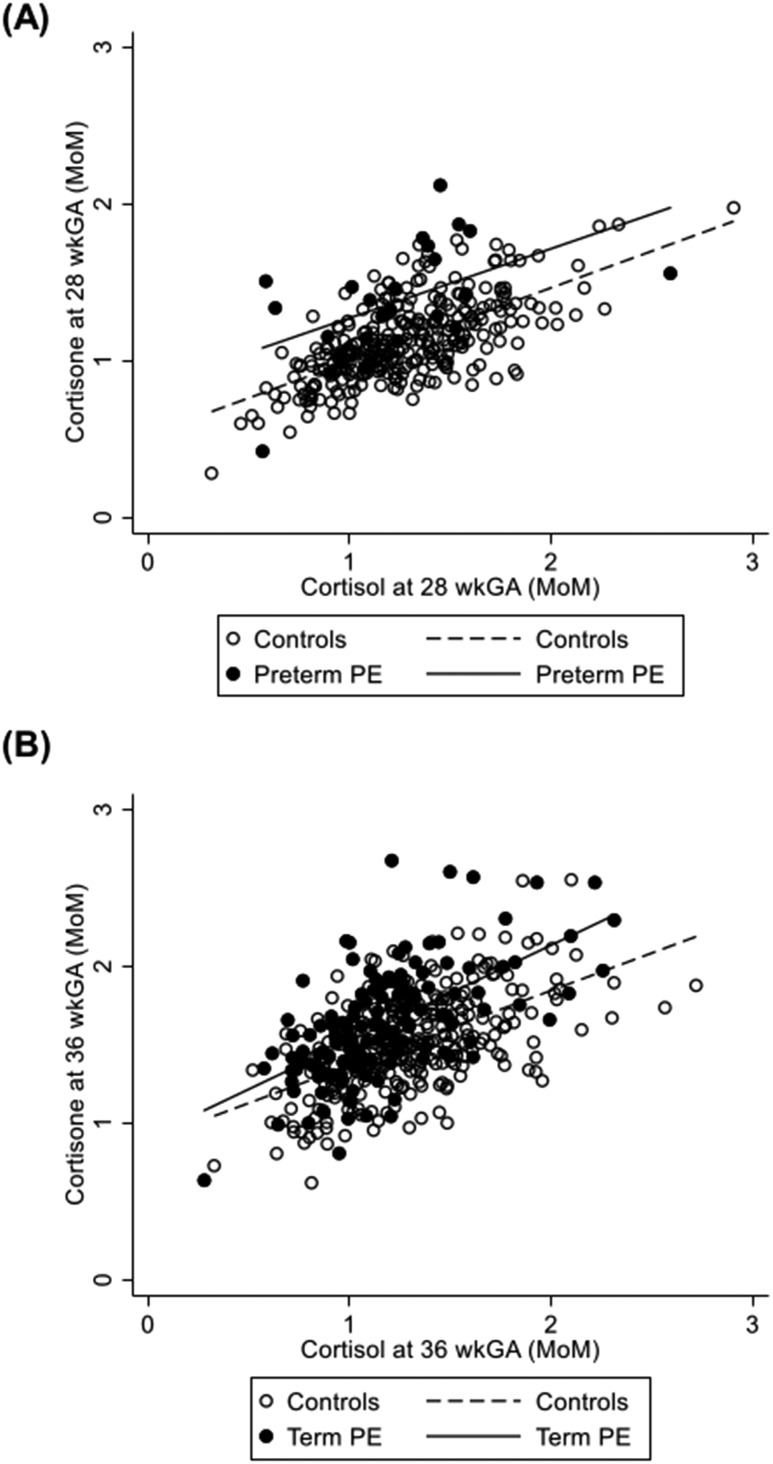
Scatter plots for cortisol vs cortisone at (A) 28 wkGA in cases of preterm PE and controls and (B) 36 wkGA in cases of term PE and controls. Cortisol and cortisone are reported as MoM. Cases of PE were defined by the ACOG 2013 Guidelines (25) and included all severe and nonsevere, nonsuperimposed PE at term and all preterm PE. Controls included women who did not have PE. Preterm delivery included pregnancies delivered <37 wkGA, and term delivery included pregnancies delivered ≥37 wkGA. For the measurement at 28 wkGA, the equation for the regression line in cases of preterm PE is *y* = 0.829 + 0.442 ⋅ *x* (*P* value for association = 0.01) and in controls, is *y* = 0.526 + 0.469 ⋅ *x* (*P* value for association < 0.001). The *P* value for interaction between preterm PE cases and controls at 28 wkGA is 0.80. For the measurement at 36 wkGA, the equation for the regression line in cases of term PE is *y* = 0.909 + 0.612 ⋅ *x* (*P* value for association <0.001) and in controls is *y* = 0.887 + 0.479 ⋅ *x* (*P* value for association < 0.001). The *P* value for interaction between term PE cases and controls at 36 wkGA is 0.10.

**Figure 3.**
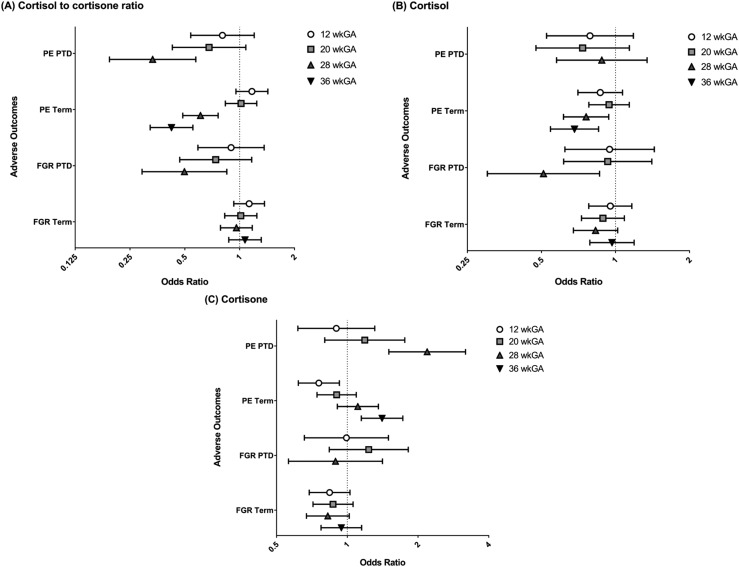
Unadjusted ORs with 95% CIs at 12, 20, 28, and 36 wkGA for term PE, preterm PE, term FGR, and preterm FGR for (A) cortisol-to-cortisone ratio, (B) cortisol, and (C) cortisone. ORs are for cases referent to controls, associated with a 1-SD higher cortisol-to-cortisone ratio, cortisol, or cortisone value. Error bars represent the 95% CIs. Cases of PE were defined by the ACOG 2013 Guidelines (25) and included all severe and nonsevere, nonsuperimposed PE at term and all preterm PE. Cases of term FGR included severe SGA (birth weight less than third customized percentile at term), SGA with growth restriction (birth weight <10th customized percentile at term with reduced growth velocity of the abdominal circumference on serial ultrasound scans), and all cases of PE were excluded from analyses of term FGR. Cases of preterm FGR included SGA (birth weight <10th customized percentile) with preterm delivery, and cases of preterm PE were excluded from analyses of preterm FGR. Preterm delivery included pregnancies delivered <37 wkGA, and term delivery included pregnancies delivered ≥37 wkGA. PTD, preterm delivery.

There was a positive association between cortisol and cortisone in cases and controls, but for a given level of cortisol, the cortisone level was greater in cases of preterm PE at 28 wkGA and term PE at 36 wkGA [Fig. 2(A) and 2(B), respectively]. The cortisol-to-cortisone ratio was negatively associated with PE [Table 2 and Fig. 3(A)] at 28 and at 36 wkGA, and the association was not materially affected by adjustment for maternal characteristics (Table 2). At 28 wkGA, the cortisol-to-cortisone ratio showed a negative association with the risk of preterm PE, term PE, and preterm FGR. At 36 wkGA, the cortisol-to-cortisone ratio was also negatively associated with term PE but not with term FGR [Fig. 3(A)]. The associations were broadly similar when the analysis was restricted to women who were not treated with steroids antenatally (Table 3). The association between the cortisol-to-cortisone ratio at 28 wkGA and preterm FGR was not statistically significant at an *α* of 0.05, but there was no evidence of an interaction between the groups with and without antenatal steroid use (*P* = 0.15 for the unadjusted analysis and *P* = 0.13 for the adjusted analysis). Hence, the apparent loss of statistical significance likely reflected reduced statistical power as a result of a smaller sample size.

**Table 2.** Unadjusted and Adjusted ORs (95% CI) for PE and FGR by 1 SD Higher Cortisol-to-Cortisone Ratio Measured at Different Time Points in Pregnancy

Approximate Gestation of Measurement, wkGA	PE^*a*^				FGR^*b*^			
	Preterm		Term		Preterm		Term	
	Unadjusted OR (95% CI)	Adjusted OR^*c*^ (95% CI)	Unadjusted OR (95% CI)	Adjusted OR^*c*^ (95% CI)	Unadjusted OR (95% CI)	Adjusted OR^*c*^ (95% CI)	Unadjusted OR (95% CI)	Adjusted OR^*c*^ (95% CI)
12	0.80 (0.54–1.20) n/N *=* 29/331	0.68 (0.44–1.04) n/N = 29/331	1.17 (0.95–1.43) n/N = 150/425	1.14 (0.92–1.41) n/N = 150/425	0.90 (0.59–1.36) n/N = 25/327	0.93 (0.60–1.45) n/N = 25/327	1.13 (0.93–1.37) n/N = 156/418	1.15 (0.94–1.41) n/N = 156/418
20	0.68 (0.43–1.08) n/N = 25/331	0.74 (0.46–1.21) n/N = 25/331	1.02 (0.83–1.24) n/N = 152/433	1.09 (0.88–1.34) n/N = 152/433	0.74 (0.47–1.16) n/N = 25/331	0.78 (0.49–1.24) n/N = 25/331	1.01 (0.83–1.24) n/N = 156/425	1.09 (0.88–1.35) n/N = 156/425
28	0.33 (0.19–0.57)^*d*^ n/N = 24/328	0.34 (0.19–0.61)^*d*^ n/N = 24/328	0.61 (0.49–0.76)^*d*^ n/N = 151/430	0.61 (0.49–0.77)^*d*^ n/N = 151/430	0.50 (0.29–0.85)^*e*^ n/N = 21/325	0.50 (0.29–0.85)^*e*^ n/N = 21/325	0.96 (0.79–1.17) n/N = 154/420	0.96 (0.78–1.19) n/N = 154/420
36^*f*^			0.42 (0.32–0.55)^*d*^ n/N = 134/409	0.42 (0.32–0.56)^*d*^ n/N = 134/409			1.07 (0.87–1.31) n/N = 148/410	1.14 (0.92–1.41) n/N = 148/410

^a^ Diagnosed according to ACOG Guidelines (25) and divided into preterm (delivery <37 wkGA) and term (delivery ≥37 wkGA) outcomes. Cases of PE include all severe and nonsuperimposed, nonsevere PE at term, and all preterm PE.

^b^ Divided into preterm (delivery <37 wkGA) and term (delivery ≥37 wkGA) outcomes. Cases of FGR at term include severe SGA (birth weight less than third customized percentile) and SGA with growth restriction (birth weight <10th customized percentile and reduced growth velocity of the abdominal circumference on serial ultrasound scans) without PE. Cases of preterm FGR include SGA (birth weight <10th percentile) with preterm delivery and without PE.

^c^ ORs adjusted for antenatal height, age, BMI, marital status, ethnicity, smoking, age at leaving FTE, and deprivation. In the analysis of preterm PE, the adjustments for ethnicity and smoking were omitted, as these variables predicted the outcome perfectly.

^d^ *P* < 0.001.

^e^ *P* < 0.05.

^f^ The 36-wkGA measurements have not been analyzed for preterm outcomes.

**Table 3.** Unadjusted and Adjusted ORs (95% CI) for PE and FGR by 1 SD Higher Cortisol-to-Cortisone Ratio Measured at Different Time Points in Pregnancy Confined to Women Who Were Documented As Not Having Received Steroids Antenatally

Approximate Gestation of Measurement, wkGA	PE^*a*^				FGR^*b*^			
	Preterm		Term		Preterm		Term	
	Unadjusted OR (95% CI)	Adjusted OR^*c*^ (95% CI)	Unadjusted OR (95% CI)	Adjusted OR^*c*^ (95% CI)	Unadjusted OR (95% CI)	Adjusted OR^*c*^ (95% CI)	Unadjusted OR (95% CI)	Adjusted OR^*c*^ (95% CI)
12	0.70 (0.37–1.33) n/N = 12/248	0.57 (0.28–1.16) n/N = 12/248	1.15 (0.91–1.44) n/N = 113/331	1.12 (0.88–1.43) n/N = 113/331	1.00 (0.59–1.68) n/N = 15/251	0.97 (0.56–1.69) n/N = 15/251	1.15 (0.93–1.42) n/N = 120/328	1.13 (0.90–1.42) n/N = 120/328
20	0.59 (0.26–1.32) n/N = 9/253	0.59 (0.25–1.41) n/N = 9/253	1.02 (0.81–1.29) n/N = 113/339	1.06 (0.84–1.35) n/N = 113/339	0.91 (0.52–1.61) n/N = 15/259	0.95 (0.52–1.73) n/N = 15/259	1.00 (0.79–1.26) n/N = 119/335	1.06 (0.83–1.36) n/N = 119/335
28	0.22 (0.08–0.66)^*e*^ n/N = 9/246	0.10 (0.02–0.51)^*e*^ n/N = 9/246	0.54 (0.41–0.71)^*d*^ n/N = 112/330	0.54 (0.41–0.71)^*d*^ n/N = 112/330	0.71 (0.37–1.35) n/N = 12/249	0.71 (0.35–1.41) n/N = 12/249	1.00 (0.80–1.26) n/N = 117/325	0.98 (0.77–1.25) n/N = 117/325
36^*f*^			0.42 (0.31–0.57)^*d*^ n/N = 99/315	0.41 (0.30–0.57)^*d*^ n/N = 99/315			1.09 (0.86–1.39) n/N = 114/320	1.14 (0.89–1.47) n/N = 114/320

^a^ Diagnosed according to ACOG Guidelines (24) and divided into preterm (delivery <37 wkGA) and term (delivery ≥37 wkGA) outcomes. Cases of PE included all severe and nonsuperimposed, nonsevere PE.

^b^ Divided into preterm (delivery <37 wkGA) and term (delivery ≥37 wkGA) outcomes. Cases of FGR at term include severe SGA (birth weight less than third customized percentile) and SGA with growth restriction (birth weight <10th customized percentile and reduced growth velocity of the abdominal circumference on serial ultrasound scans) without PE. Cases of preterm FGR include SGA (birth weight <10th percentile) with preterm delivery and without PE.

^c^ ORs adjusted for antenatal height, age, BMI, marital status, ethnicity, smoking, age at leaving FTE, and deprivation. In the analysis of preterm PE, the adjustments for ethnicity and smoking were omitted, as these variables predicted the outcome perfectly.

^d^ *P* < 0.001

^e^ *P* < 0.05

^f^ The 36-wkGA measurements have not been analyzed for preterm outcomes.

Cortisol was negatively associated with the risk of term PE from 28 wkGA onward [Fig. 3(B)], whereas the association between cortisone and the risk of term PE was negative in the first trimester and changed to positive in the third trimester [Fig. 3(C)]. A similar association was also seen with cortisone and preterm PE [Fig. 3(C)] but not cortisol [Fig. 3(B)]. The associations seen were stronger as pregnancy progressed for both the cortisol-to-cortisone ratio and cortisone in term and preterm PE [Fig. 3(A) and 3(C)]. Cortisol was negatively associated with preterm FGR at 28 wkGA [Fig. 3(B)], but there were no clear associations between cortisone and preterm FGR [Fig. 3(C)], and there was no clear association between cortisol or cortisone and term FGR [Fig. 3(B) and 3(C)].

### Cumulative incidence of PE according to cortisol-to-cortisone ratio

**Figure 4.**
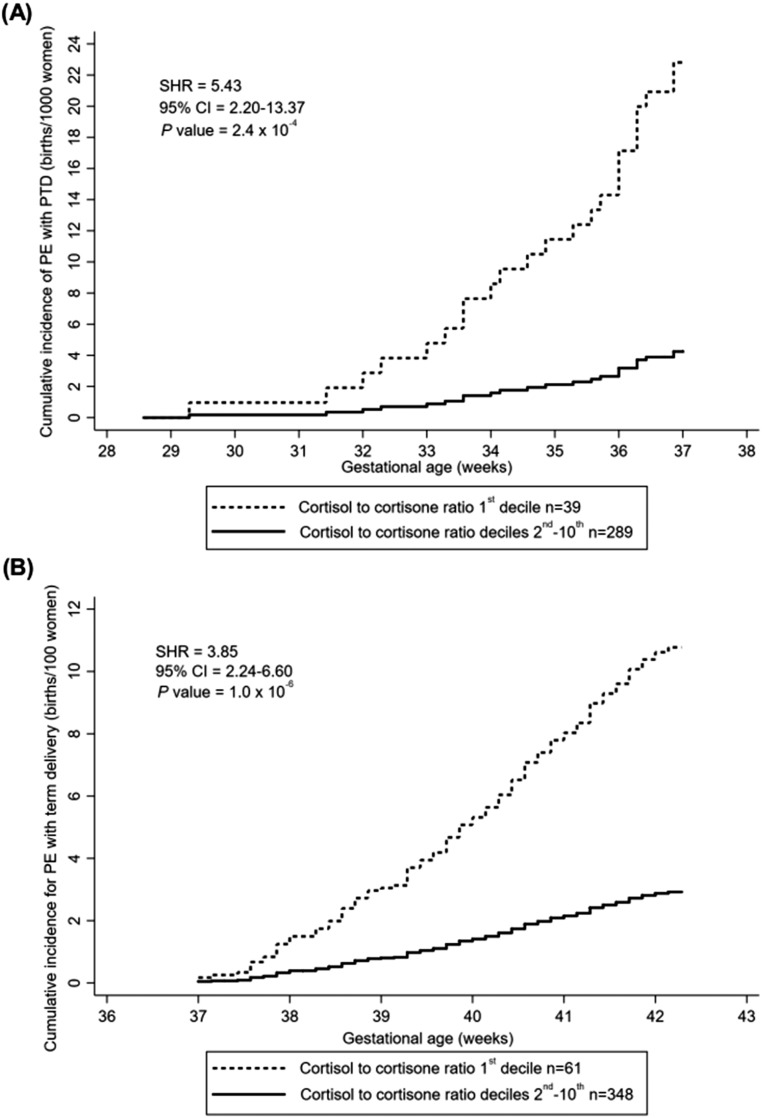
Cumulative incidence of PE comparing the first decile of the cortisol-to-cortisone ratio with the second through 10th deciles of cortisol-to-cortisone ratio from (A) 28 wkGA onward in cases of preterm PE and (B) 36 wkGA onward in cases of term PE. (A) Reported in births per 1000 women; (B) reported in births per 100 women. Cases of PE were defined by the ACOG 2013 Guidelines (25) and included all severe and nonsevere, nonsuperimposed PE at term and all preterm PE. Preterm delivery included pregnancies delivered <37 wkGA, and term delivery included pregnancies delivered ≥37 wkGA. Delivery without PE was considered as competing risk. PTD, preterm delivery; SHR, subhazard ratio.

There was a higher cumulative incidence of preterm PE and term PE for women in the lowest decile of the cortisol-to-cortisone ratio compared with deciles two to 10 at the 28- and 36-wkGA measurements, respectively; ∼2.2% and 11% cases in the lowest decile vs 0.4% and 3% in deciles two to 10. For preterm PE, the curves started to deviate 2 weeks after the 28-wkGA measurement [Fig. 4(A)], and for term PE, the curves began to deviate at least 1 week after the 36 wkGA measurement [Fig. 4(B)]. Similar results were obtained when women treated with steroids antenatally were excluded.

## Discussion

In this study, we found a clear negative association between the maternal serum cortisol-to-cortisone ratio and the risk of preterm and term PE. In women with term PE, the cortisol-to-cortisone ratio was significantly lower >8 weeks before delivery. In term PE, cortisol levels were lower at 28 wkGA, and cortisone levels were higher at 36 wkGA, and in preterm PE cortisone levels were higher at 28 wkGA. These changes could represent higher 11*β*HSD2 activity or lower 11*β*HSD1 activity in women with PE, which occur before the manifestation of clinical signs and symptoms.

Multiple studies have shown lower placental 11*β*HSD2 activity after vaginal and cesarean delivery in women with PE and FGR (7–15), but our work and that of other studies investigating maternal blood and urine cortisol-to-cortisone ratio in women with established disease (18–20) suggest that maternal systemic 11*β*HSD2 activity is increased. The observation of reduced 11*β*HSD2 activity in placentas from women with PE may reflect general placental failure and local regulation of cortisol metabolism by the feto-placental unit, which is independent of the mother. Aufdenblatten *et al.* (31) found that placental cortisol was almost completely inactivated in normotensive pregnancies, indicating an effective 11*β*HSD2 barrier from high levels of cortisol. This was in contrast to pregnancies with PE and low birth weight that had reduced placental 11*β*HSD2 activity and increased placental cortisol levels (32). Therefore, as 11*β*HSD2 is also expressed outside of the placenta, the association between PE and FGR with a lower cortisol-to-cortisone ratio could be related to extraplacental 11*β*HSD2 activity, such as maternal renal 11*β*HSD2 (33, 34).

PE is frequently accompanied by renal dysfunction, but impaired renal function and hypertension in diseases outside of pregnancy have been associated with decreased renal 11*β*HSD2 expression and activity (31, 35). Thus, maternal 11*β*HSD2 activity may increase as a compensatory response in PE, acting to reduce systemic cortisol. The systemic stress and inflammatory response that occur in PE (36, 37) may result in higher maternal cortisol secretion, which would suggest that 11*β*HSD2 activity increases to the extent that conversion of cortisol-to-cortisone exceeds cortisol secretion. However, cortisol secretion is not definitively known to rise in PE, and there is limited evidence that cortisol itself stimulates 11*β*HSD2 activity; exogenous corticosteroids have been shown to stimulate 11*β*HSD2 activity in bronchial epithelial cells (38), but the effects of cortisol on renal 11*β*HSD2 activity are not known, and as we observed a linear relationship between cortisol and cortisone levels in women with and without PE, it is likely that there are other factors responsible for stimulating 11*β*HSD2 action.

Another explanation for the changes observed in cortisol and cortisone levels might be reduced 11*β*HSD1 activity, as opposed to increased 11*β*HSD2 activity. There are studies showing reduced placental and chorionic 11*β*HSD1 activity in infants born SGA (birth weight <10th percentile) (10, 16) that could be a compensatory mechanism to reduce fetal cortisol exposure as a result of increased placental crossover of cortisol. We have been unable to identify any studies on the activity of placental 11BHSD1 from women with PE. Furthermore, 11*β*HSD1 is present in the chorio-decidua (39, 40), but it is unclear whether reduced 11*β*HSD1 activity at the feto-maternal interface would affect maternal cortisol and cortisone levels. Reduced maternal systemic 11*β*HSD1 activity is another possible explanation for the findings, but this is also yet to be investigated and would be an important area of future research.

Our study had several methodological strengths. First, this study looks at measurements of cortisol and cortisone in maternal serum preceding the onset of disease in both PE and FGR. Second, the case-cohort study design meant that we were able to study a large number of cases of PE and FGR. Moreover, as a result of the large size of the cohort and the availability of serial ultrasound scans, we were able to confine our analysis of FGR to either infants who were extremely SGA (less that third percentile) or infants <10th percentile with other features, indicating a likely pathological cause (preterm birth or reduced fetal growth velocity); many other studies have only studied SGA infants (<10th percentile), and a large proportion of these will be healthy. The case-cohort design also meant that we were able to compare cases of PE and FGR with a population representative of the whole cohort. This is more likely to demonstrate a true difference in participants, with and without the outcomes studied, than a case-control study; in case-control studies, the comparison group is often healthy, and differences seen may only reflect the lack of other outcomes in the control group rather than the outcome of interest in cases. Details of the methodology underlying case-cohort studies and potential statistical and reporting issues are described by Sharp *et al.* (41).

However, our study also has some limitations. Cortisol and cortisone were quantified as MoM rather than (SI) Units, so values cannot be directly compared with traditional measures of cortisol and cortisone in nanomoles per liter. This is because the metabolomics assay was untargeted, and internal standards were not available for all metabolites. However, the quantification method is accurate, and the ratio is unitless, so the results correctly reflect cortisol and cortisone levels measured using standard methods. Additionally, human cortisol secretion follows a circadian rhythm, which was not accounted for in this study (42). However, Kosicka *et al.* (19) controlled for diurnal variation by taking early-morning blood samples and drew the same conclusion that the maternal serum cortisol-to-cortisone ratio was reduced in PE. Cortisol secretion is influenced by external factors, including stress and anxiety (43). Although we adjusted for some characteristics, such as deprivation and marital and smoking status, it was difficult to quantify any other external stresses that the study participants may have been experiencing. However, confounding by maternal stress is unlikely to have caused the associations, as it would tend to be associated with increased rather than decreased cortisol levels. Furthermore, we also considered whether our findings could have been influenced by exogenous antenatal steroid use, and there were no clear differences in our results when these women were excluded. Whereas the association between the cortisol-to-cortisone ratio and preterm FGR at 28 wkGA, where cases of antenatal steroid use had been excluded, was not significant at an *α* of 0.05, the point estimates (OR) were within the 95% CIs of the analyses where they had not been excluded, and there was no evidence of an interaction between the ratio and exposure to steroids; thus, the higher *P* values reflected reduced statistical power from a smaller sample size.

In conclusion, this study demonstrates that a lower maternal serum cortisol-to-cortisone ratio precedes the clinical manifestation of PE and preterm FGR by many weeks, despite previous reports of elevated levels of placental 11*β*HSD2 in these conditions. Our observations implicate enhanced maternal 11*β*HSD2 activity in the pathophysiology of PE. However, further investigation into the potential use of the cortisol-to-cortisone ratio as a predictive marker of PE would be beneficial, given that there is no current accurate predictive test for the condition. In addition, research into maternal and placental 11*β*HSD1 and 11*β*HSD2 activity in women with PE and FGR, and links to fetal and newborn cortisol metabolism would be useful in gaining a deeper understanding of the pathophysiology of the diseases.

## Abbreviations:

11*β*HSD1: 11-*β*-hydroxysteroid dehydrogenase type 1
11*β*HSD2: 11-*β*-hydroxysteroid dehydrogenase type 2
ACOG: American College of Obstetricians and Gynecologists
BMI: body mass index
FGR: fetal growth restriction
FTE: full-time education
MoM: multiples of the median
PE: preeclampsia
POP: Pregnancy Outcome Prediction
SGA: small for gestational age
wkGA: weeks of gestational age
